# Quinoid‐Resonant Conducting Polymers Achieve High Electrical Conductivity over 4000 S cm^−1^ for Thermoelectrics

**DOI:** 10.1002/advs.201800947

**Published:** 2018-08-23

**Authors:** Dafei Yuan, Liyao Liu, Xuechen Jiao, Ye Zou, Christopher R. McNeill, Wei Xu, Xiaozhang Zhu, Daoben Zhu

**Affiliations:** ^1^ Beijing National Laboratory for Molecular Sciences CAS Key Laboratory of Organic Solids Institute of Chemistry Chinese Academy of Sciences Beijing 100190 P. R. China; ^2^ School of Chemistry and Chemical Engineering University of Chinese Academy of Sciences Beijing 100049 P. R. China; ^3^ Department of Materials Science and Engineering Monash University Clayton Victoria 3800 Australia; ^4^ Australian Synchrotron ANSTO 800 Blackburn Rd Clayton VIC 3168 Australia

**Keywords:** conducting polymers, conductivity, organic thermoelectrics, phase transformation, quinoid resonant

## Abstract

New conducting polymers polythieno[3,4‐*b*]thiophene‐Tosylate (PT*b*T‐Tos) are prepared by solution casting polymerization. Through tuning the alkyl group of T*b*T, the electrical conductivity can be effectively enhanced from 0.0001 to 450 S cm^−1^. Interestingly, the electrical conductivity of PT*b*T‐C1‐Tos increases significantly from 450 S cm^−1^ at room temperature to 4444 S cm^−1^ at 370 K, which is disparate from polyethylenedioxythiophene‐Tos exhibiting metallic conducting behavior. Quasi‐reversible phase transformation with temperature from 3D crystallites to lamellar‐stacking coincides with the increasing electrical conductivity of PT*b*T‐C1‐Tos with heating. Methyl‐substituted PT*b*T‐Tos with the best electrical property is further utilized for thermoelectrics and a power factor as high as 263 µW m^−1^ K^−1^ is obtained. It is believed that PT*b*T‐Tos will be a promising family of conducting polymers for solution‐processed organic electronics.

Conducting polymers have received great attentions since the pioneering works by Alan MacDiarmid, Hideki Shirakawa, and Alan J. Heeger, who found that serendipitous polyacetylene shows metallic conducting property after doped by iodine.^1^ These new generation of polymers featuring conjugated backbones combine both the chemical and the mechanical properties of the plastics and the metallic conducting behavior. In the recent several decades, polyacetylene, polythiophene, polypyrrole, polyaniline (PANI), polyparaphenylene vinylene, polyethylenedioxythiophene (PEDOT), etc. have been developed and their conducting property were investigated systematically by chemical or electrochemical doping.^2^ Moreover, these polymer conductors have shown great prospect for next generation of optoelectronic devices including organic light‐emitting diodes, optical transparent electrodes, ion‐storage layer, etc.^3^ Recently, conducting polymers have been successful employed in thermoelectric applications because of their intrinsically low thermal conductivity and high electrical conductivity.^4^

Because of the unique quinoid‐enhancing effect^5^, ^6^ (**Scheme** **1**), the ring‐fused polythiophene derivatives including PITN, PTP, polythieno[3,4‐*b*]thiophene (PT*b*T), etc. can achieve both narrowed energy bandgaps and enhanced π‐conjugation.^7^ After doping, the induced charge carriers can transport smoothly on these conjugated backbones, from which a high electrical conductivity may be achieved as the charge carriers can be stabilized by the quinoidization effect. The family of polymers have been developed since 1997.^7^ The first soluble PT*b*T was synthesized by Pomerantz et al. through oxidation polymerization of T*b*T monomers by FeCl_3_, which led to a low optical bandgap of 0.92 eV and electrical conductivity of 0.0031 S cm^−1^ after doped by FeCl_3_.^8^ Neef et al. developed phenyl substituted PT*b*T by electrochemical polymerization with a more narrowed bandgap of 0.85 eV obtained.^9^ PT*b*T polymers without alkyl group were developed by Sotzing and Lee through electrochemical polymerization of the T*b*T monomer^10^ and dimers,^11^ which showed high stability for both n‐type and p‐type doping. Because of more than two reaction sites on these monomers, network‐like homo–polymers were formed. The conductivity of these PT*b*T homo–polymers were determined to be 10^−5^ S cm^−1^ in the neutral state, which increased to 0.2 S cm^−1^ by four orders of magnitude in the doped state. The water dispersion PT*b*T‐PSS were also synthesized by the same group,^12^ with drop‐cast films showing a conductivity of 0.02 S cm^−1^ with a bandgap lower than 1.0 eV. Bendikov and co‐workers developed a solid‐state polymerization method^13^ by directly heating T*b*T‐2Br monomer on a hot‐plate for more controlled polymerization. Pressed pellets of PT*b*T showed an electrical conductivity of 6 S cm^−1^ with a bandgap of 0.96 eV. To date, the low electrical conductivity of doped PT*b*Ts makes it quite difficult for practical applications.^14^

**Scheme 1:**
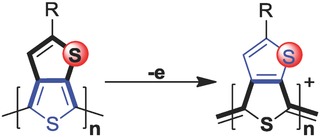
Quinoid‐enhancing effect facilitates doping.

To achieve high electrical conductivity for doped PT*b*Ts, linear polymerization and high doping level is essential. In this work, a new family of doped PT*b*Ts, namely PT*b*T‐Tos are prepared by in situ solution casting polymerization.^15^, ^16^ Through fine tuning the alkyl group at the two‐position of T*b*T, the electrical conductivities can be significantly enhanced from 0.0001 (*n*‐octyl) to 450 (methyl) S cm^−1^. Disparate from the metallic property of PEDOT‐Tos,^15^ the electrical conductivity of PT*b*T‐C1‐Tos enhances over one order of magnitude with elevated temperature to the highest value of 4444 S cm^−1^. Methyl‐substituted PT*b*T‐Tos with the best electrical property is further utilized for thermoelectrics, resulting in a power factor as high as 263 µW m^−1^ K^−1^.

T*b*T monomers with different alkyl substitutions are synthesized following the reported procedures.^6^, ^13^ For convenience, C8, C6, C4, C3, C1, and C0 are used as shorthand expressions for the alkyl groups C_8_H_17_, C_6_H_13_, C_4_H_9_, C_3_H_7_, CH_3_, and H, respectively (**Scheme** **2**). After mixing the T*b*T monomers with the Baytron C solutions,^16^, ^17^ the mixtures are spin‐coated onto glass substrates and then transferred to a hot‐plate to polymerize at an optimized temperature of 90 °C under ambient conditions for 2 h. The polymer films are then washed with ethanol carefully, and dry on the hot‐plate at 90 °C for half an hour (optimized procedures are shown in Figure S1 of the Supporting Information in detail). The thickness of the prepared films is determined to be around 200–250 nm by profilometer.

**Scheme 2:**
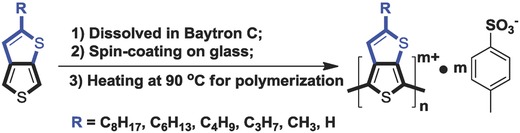
Preparation of conducting polymers, PT*b*T‐Tos.

The standard four‐probe method is used to determine the electrical conductivities of PT*b*T‐Tos films. As shown in **Figure** **1**a, the average electrical conductivities are significantly enhanced from 0.0001 to 450 S cm^−1^ from PT*b*T‐C8‐Tos to PT*b*T‐C1‐Tos with shortening of the alkyl group. However, the electrical conductivity decreases to 300 S cm^−1^ for PT*b*T‐C0‐Tos because of a relatively disordered cross‐linked network.^10^, ^11^ Temperature‐dependent electrical conductivity is determined in situ by placing the PT*b*T‐C1‐Tos films on hot‐plate. The electrical conductivity is significantly increased from 450 S cm^−1^ at 300 K to the highest value of 4444 S cm^−1^ at 370 K (Figure 1b). The electrical conductivity of the PT*b*T‐C1‐Tos films can be recovered when the temperature is returned to room temperature (Figure 1c), indicating a highly temperature‐dependent behavior and is contrary to what is observed for PEDOT‐Tos[[qv: 15a]] (Figure 1b), whose electrical conductivities decreased gradually with elevated temperature from 600 to 450 S cm^−1^. Even though various conducting polymers have been developed for decades, only two cases: polyaniline[[qv: 15b]] and PEDOT‐Tos[[qv: 15a]] are found to exhibit metallic behavior. One possible reason might be related to the high degree of structural order and less crystal lattice defect in PANI and PEDOT‐Tos, which lead to the intrinsic metallic conductivity with temperature.^20^ However, most conducting polymers show thermal‐activated hopping behavior such as PEDOT‐PSS and PT*b*T‐Tos in this work because of the amorphous character or relatively disordered polymer aggregates. Meanwhile, different from the absence of ESR response for PEDOT‐Tos with bipolaron transport, the PT*b*T‐C1‐Tos film exhibits a strong ESR signal (Figure 1d) at *g* = 2.00643, possibly indicating polaron‐dominant transport mode.

**Figure 1:**
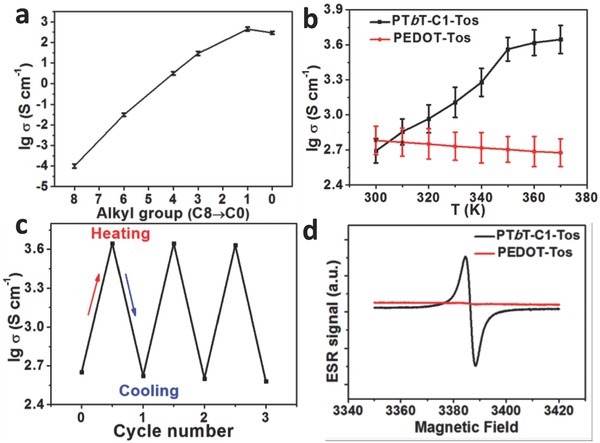
a) The electrical conductivity (logarithm) of PT*b*T‐Tos with alkyl groups from C8 to C0. b) Electrical conductivity (logarithm) of PT*b*T‐C1‐Tos and PEDOT‐Tos changes with elevated temperature. c) Electrical conductivity of PT*b*T‐C1‐Tos film with heating and cooling (three cycles). d) ESR spectra of PT*b*T‐C1‐Tos and PEDOT‐Tos films.

According to the equation: σ = *nµq*, where σ is electrical conductivity, *n* is carrier concentration, *µ* is charge mobility, *q* is carrier charge, electrical conductivity (σ) is determined by the charge transport ability (*µ*) and doping levels (*n*). Ultraviolet–visible near‐infrared spectroscopy (UV–vis–NIR), ultraviolet photoelectron spectroscopy (UPS), and X‐ray photoelectron spectroscopy (XPS) measurements are performed to investigate doping level of PT*b*T‐Tos with different alkyl groups in thin films. As shown in the UV–vis–NIR spectra (**Figure** **2**a), the absorption peaks at around 500–1500 nm disappear gradually from PT*b*T‐C8‐Tos to PT*b*T‐C1‐Tos, being replaced by wide charge‐resonance band extending over 2500 nm. The increased absorption bands imply enhanced doping level (oxidation process) with shortening of alkyl chain from C8 to C1. The closure of bandgap in PT*b*T‐C1‐Tos, similar to the metal absorption, should facilitate the generation of free carriers by thermal activation and charge transport.^18^ The S(2p) peak at the binding energy of 166–170 eV in XPS spectra is attributed to the sulfur of Tos^–^ that is coupled with the positive charge on PT*b*T chain,^16^, ^17^ representing the doping levels. As shown in Figure 2b and Figure S4 (Supporting Information), the intensity of this sulfur peak of Tos^−^ increases gradually with shortening of the alkyl chain length together with the binding energy near 164 eV assigned to sulfur on T*b*T shifting toward lower value, which is consistent with the increasing doping levels. On the other hand, the doping induced charged T*b*T units shifts the most intense peak near 164 eV (Sulfur in PT*b*T) toward lower binding energy, in accordance with the shifts of the vacuum level and valence band spectra as discussed below due to the band bending effect, also suggests a higher doping level with decreasing length of the alkyl substituent. From the UPS data, the work function is found to significantly shift from 4.04 eV for PT*b*T‐C6‐Tos downward to 4.50 eV for PT*b*T‐C1‐Tos as depicted in low kinetic energy region (Figure 2c). Meanwhile, the binding energy as shown in the highest occupied molecular orbital (HOMO) onset region in the UPS spectra (Figure 2d; Figure S5, Supporting Information) is significantly shifting toward lower values going from 0.38 (C8) to ≈0 eV (C1). Moreover, the HOMO onset binding energy extending past zero as shown for PT*b*T‐C1‐Tos and PT*b*T‐C0‐Tos, imply that the Fermi level is overlapping with the HOMO energy level, facilitating the generation of carriers and consistent with the UV–vis–NIR absorption. Among them, the doping level of PT*b*T‐C1‐Tos with the shortest alkyl chain is the highest as concluded from the strongest S(2p) peaks in the XPS spectra and the most significant down‐shift of Fermi level from the UPS spectra. As a result, we could deduce that the nonpolarity nature and steric hindrance of alkyl groups on T*b*T units might restrain the approaching of the oxidant, Fe(Tos)_3_, to T*b*T and affect oxidative polymerization, which lead to lower doping levels for the PT*b*T‐Tos polymers with longer alkyl chains. In comparison, the doping level of PT*b*T‐C0‐Tos without alkyl group is slightly higher than PT*b*T‐C1‐Tos as shown in Figures S4 and S5 of the Supporting Information. The increased doping level is consistent with the enhanced electrical conductivity going from PT*b*T‐C8‐Tos to PT*b*T‐C1‐Tos and PT*b*T‐C0‐Tos. Moreover, from the HOMO onset of PT*b*T‐C1‐Tos in UPS spectra (Figure 2d), the small slope of binding energy extending over zero is similar to PEDOT‐PSS.^15^ Together with the same strong ESR signal and electrical conductivity temperature‐dependent behavior, the dominant charge carriers in PT*b*T‐C1‐Tos might be similar to the PEDOT‐PSS (Polaron‐dominant). As a result, the increased electrical conductivity with elevating temperature in PT*b*T‐C1 may be partially attributed to the thermally activation hopping mechanism normally found in conducting polymers.^19^, ^20^

**Figure 2:**
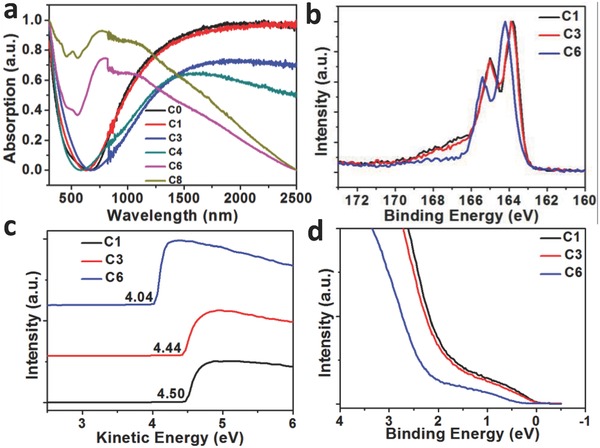
a) The UV–vis‐NIR absorption spectra of PT*b*T‐Tos with alkyl groups from C8 to C0. b) S(2p) XPS spectra of The PT*b*T‐Tos polymers with alkyl group C6, C3, and C1. c) The low kinetic energy region and d) the low binding energy region (HOMO) of UPS spectra.

To explore the effect of the alkyl chain length on microstructures and charge transport in PT*b*T‐Tos films, grazing incidence wide‐angle X‐ray scattering (GIWAXS) and scanning electron microscopy (SEM) measurements are conducted. The lamellar stacking distance is well resolved by the GIWAXS measurements (**Figure** **3**c; Figure S3, Supporting Information). The low *q* peaks below *q* = 0.5 Å^−1^ correlate well with the side‐chain lengths. Increasing the side chain length obviously increase the lamellar stacking distance, evidenced by the reduced *q* value. The longer insulating alkyl groups inserting between polymer backbones are believed to influence the charge transport via its impact on the packing behaviors along the π‐stacking direction and backbone direction. Intriguingly, the 1D GIWAXS profile of PT*b*T with the shortest alkyl length namely C1, show weak and broad peaks across the whole *q* range, whereas other PT*b*T derivatives exhibit well‐defined peaks, including the lamellar stacking peaks at *q* < 0.5 Å^−1^, and 3D crystalline peaks ranging from *q* = 1 Å^−1^ to *q* = 2 Å^−1^. This observation may imply that the alkyl side chain with insufficient length is not capable of inducing highly ordered molecular arrangement.^21^, ^22^ Although PT*b*T‐C1‐Tos exhibits the least ordered edge‐on lamellar crystallites, evidenced by the weak out‐of‐plane lamellar peak, it is seen that the PT*b*T‐C1‐Tos film has the highest electrical conductivity, higher than the other PT*b*T‐Tos derivatives with alkyl side chains longer than C1. This suggests that morphological features other than the nanoscopic crystallization and aggregation may impose noticeable impact on the electrical properties. To this end, the mesoscopic topological information is probed by the SEM measurements and it is found that with longer alkyl chains larger surface domain is imaged (Figure 3a,b; Figure S2, Supporting Information), consistent with the trend observed from the GIWAXS results.

**Figure 3:**
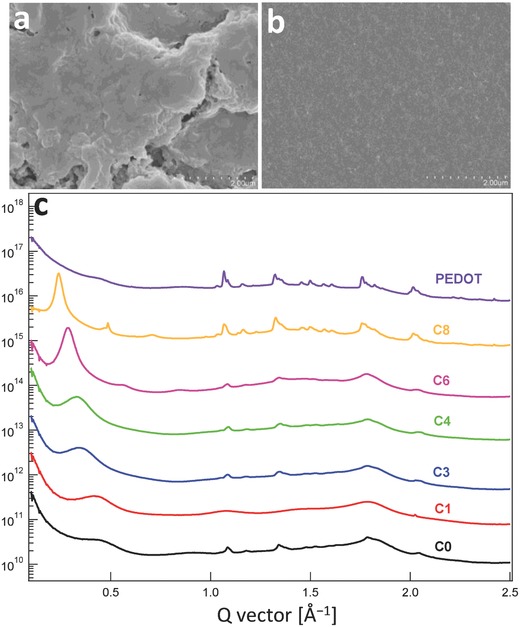
SEM (bar: 2 µm) images of a) PT*b*T‐C6‐Tos and b) PT*b*T‐C1‐Tos. c) 1D GIWAXS circular average profiles of PT*b*T‐Tos with alkyl groups from C8 to C0 and PEDOT.

Polymer's morphology changes with temperature would greatly influence charge transport in the polymer backbone. To explore the potential morphology changes with heating, temperature dependent XRD measurements are conducted on drop‐cast films of PT*b*T‐C1‐Tos. As shown in the XRD profiles (**Figure** **4**a; Figure S7, Supporting Information), the as‐cast (black line) and annealed samples (red line) generate distinctively different diffractograms. The different peak positions and widths between the as‐cast film and the annealed film suggest vastly different molecular packing patterns between the samples without and with thermal annealing. More specifically, Phase I (that predominantly exists at room temperature) exhibits intense and sharp diffraction peaks at high *q* range over *q* = 0.5 Å^−1^ with a weak but discernable lamellar diffraction at *q* = 0.33 Å^−1^, corresponding to the lamellar spacing of 19.03 Å. This implies that the PT*b*T‐C1‐Tos molecules tend to form 3D crystallites with weak lamellar stacking. With thermal annealing, Phase I rapidly disappears, indicated by the elimination of sharp peaks at 1 < *q* < 1.5 Å^−1^ and broader diffraction peaks at 1 < *q* < 1.5 Å^−1^ along with a much stronger lamellar stacking peak at *q* = 0.33 Å^−1^. Notably, the thermal annealing leads to considerably enhanced lamellar stacking, whereas the 3D feature of the PT*b*T‐C1‐Tos crystallites is observed to be highly suppressed, as evidenced by the reduced Å^−1^, accompanied by the dominance of Phase II (that predominantly exists at high temperature), which displays less prominent diffraction at the high *q* range. Intriguing, it is found that Phase I can be partially recovered when the temperature was set back to room temperature (see Figure 4a blue line; Figure S7, Supporting Information). The transformation of PT*b*T‐C1 molecular arrangement correlates well with the DSC analysis with elevating temperature (Figure 4b), which shows obviously heat absorption double peaks starting from 60 °C and ending at 130 °C. The absence of any feature during the cooling process indicates that the crystallization process in PT*b*T‐C1 is kinetically slow, which is in entire accord with the partially, rather than complete, recovery of Phase I, as evidenced by the XRD pattern recorded after returning room temperature. The temperature region of the increased electrical conductivity (Figure 1b; Figure S6a, Supporting Information), is consistent with the DSC and XRD trends. Besides, the activation energy of PTbT‐C1‐Tos calculated from Figure S6b,c of the Supporting Information, fitting to the Arrhenius equation, is determined to be 0.29 and 0.11 eV at low (300–340 K) and high temperature (350–370 K) region, respectively. In comparison to the reported highly doped conducting polymers,^20^ the relatively larger activation energy should be ascribed to the low ordered crystallites with amorphous character in PT*b*T‐C1‐Tos as concluded from GIWAXS measurements. The two different activation energies are consistent with the stacking transformation from Phase I to Phase II, which is similar to the zwitterion crystal, whose electrical conductivity has been shown to increase by nearly two orders of magnitude from 300 to 400 K.^23^ Consequently, it is believed that the molecular stacking changes and the thermal‐assisted hopping mechanism may contribute to the significantly increased electrical conductivity with elevating temperature in PT*b*T‐C1‐Tos. The quasi‐reversible transformations of electrical conductivity to near one order of magnitude and stacking mode with temperature are rare amongst reported conducting polymers, which provide a unique opportunity to understand the relationship between micro structural electrical properties.

**Figure 4:**
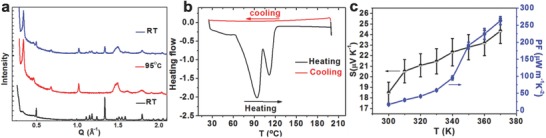
a) Temperature‐variable XRD patterns of PT*b*T‐C1‐Tos bulk film: room temperature (black line); heating to 95 °C (red line); cooling down to room temperature (blue line). b) DSC analysis and c) Seebeck coefficient and power factor of PT*b*T‐C1‐Tos with temperature.

The significantly increased electrical conductivity (σ) of the in situ PT*b*T‐C1‐Tos films with elevating temperature contributes to the enhanced thermoelectric performance following the equation: PF = *σS* ^2^. Hence, the Seebeck coefficients (*S*) of the PT*b*T‐C1‐Tos films are determined by measuring the thermovoltages after providing a temperature difference on two sides of the films (Figure S8, Supporting Information). With increasing temperature, the Seebeck coefficients is gradually increased from 18.5 to 24.4 µV K^−1^ (Figure 4c) and is reversible more than three times (Figure S9, Supporting Information). The power factor is enhanced from 13.0 µW m^−1^ K^−1^ at room temperature to 263 µW m^−1^ K^−1^ at 370 K, which is much higher than PEDOT‐Tos‐based device fabricated under the same conditions, 7.6 µW m^−1^ K^−1^ (Table S1, Supporting Information). Because of the acknowledged great challenge in the measurement of in‐plane thermal conductivity (κ_∥_) for organic thin film,^24^ κ_∥_ of PT*b*T‐C1‐Tos film cannot be obtained at the present stage. However, the amorphous character of PT*b*T‐C1‐Tos is similar to PANI^25^ and should contribute to an intrinsically low thermal conductivity, which should make PT*b*T an excellent material system for thermoelectric application (**Table** **1**).^26^, ^27^, ^28^, ^29^

**Table 1:** High‐performance organic thermoelectric materials reported recently

Materials	PF [µW m^−1^ K^−2^]	Ref.
PyDI‐5FPE‐SnCl_2_	80	26
BTBT_2_(AsF_6_)	88	27
P3HT‐TCB	62.4	28
TDPPQ‐Bi	113	29
PBTTT‐F_4_TCNQ	120	24
PT*b*T‐C1‐Tos	263	This work

In conclusion, conducting polymers PT*b*T‐Tos with different alkyl groups have been synthesized by in situ solution‐casting polymerization. The electrical conductivity can be effectively tuned ranging from 0.0001 (*n*‐octyl) to 450 (methyl) S cm^−1^ by alkyl‐chain length. The electrical conductivity of PT*b*T‐C1‐Tos enhances significantly from 450 S cm^−1^ at room temperature to 4444 S cm^−1^ at 370 K, disparate from PEDOT‐Tos with metallic conducting behavior. Quasi‐reversible phase transformation from 3D crystallites to lamellar‐stacking is found in PT*b*T‐C1‐Tos, consistent well with the significantly enhanced electrical conductivity by heating. Highly conductive PT*b*T‐C1‐Tos polymer is promising for organic electronics, which has been demonstrated by thermoelectric application.

## Conflict of Interest

The authors declare no conflict of interest.

## Supporting information

Supplementary
