# Supplementary material for: Quinoid‐Resonant Conducting Polymers Achieve High Electrical Conductivity over 4000 S cm−1 for Thermoelectrics
Source: Adv Sci (Weinh). 2018 Aug 23;5(10):1800947. doi: 10.1002/advs.201800947 (PMC6193159; doi:10.1002/advs.201800947)
Supplement: Supplementary file 1 — Supplementary [file ADVS-5-1800947-s001.pdf]

Copyright WILEY-VCH Verlag GmbH & Co. KGaA, 69469 Weinheim, Germany, 2018.

Supporting Information

for *Adv. Sci.,* DOI: 10.1002/advs.201800947

Quinoid-Resonant Conducting Polymers Achieve High Electrical Conductivity over 4000 S cm^{-1} for Thermoelectrics

Dafei Yuan, Liyao Liu, Xuechen Jiao, Ye Zou, Christopher R. McNeill, Wei Xu, Xiaozhang Zhu,* and Daoben Zhu*

Copyright WILEY-VCH Verlag GmbH & Co. KGaA, 69469 Weinheim, Germany, 2016.

Supporting Information

Quinoid-Resonant Conducting Polymers Achieve High Electrical Conductivity over 4000 S cm^{-1} for Thermoelectrics

Dafei Yuan, Liyao Liu, Xuechen Jiao, Ye Zou, Christopher R. McNeill, Wei Xu,* Xiaozhang Zhu,* and Daoben Zhu

1. General tests and experimental details

Materials. All the reactions dealing with air- or moisture-sensitive compounds were carried out in a positive atmosphere of nitrogen. Unless stated otherwise, starting materials were obtained from Adamas, Aldrich and J&K and were used without any further purification. Anhydrous THF were distilled over Na/benzophenone prior to use. The monomer thieno[3,4-*b*] thiophene (T*b*T) with alkyl group ranging from C_8H_{17} to CH_3 and H were prepared according to the published procedures. [1]

Measurements and General Methods. Hydrogen nuclear magnetic resonance (^{1}H NMR) and carbon nuclear magnetic resonance (^{13}C NMR) spectra were measured on BRUKER DMX 300 and BRUKER DMX 400 spectrometers. Chemical shifts for hydrogens are reported in parts per million (ppm, δ scale) downfield from tetramethylsilane and are referenced to the residual protons in the NMR solvent and (^{13}C NMR spectra were recorded at 100 MHz. Chemical shifts for carbons are reported in parts per million (ppm, δ scale) downfield from tetramethylsilane and are referenced to the carbon resonance of the solvent. HR-ESI and HR-MALDI-TOF measurements were performed on an Applied Biosystems 4700 Proteomics Analyzer. The Ultraviolet photoelectron spectroscopy (UPS) and X-ray photoelectron spectroscopy (XPS) measurements were carried out in a Kratos ULTRA AXIS DLD ultrahigh vacuum photoelectron spectroscopy connected to a custom-made high vacuum thermal evaporation system. The base pressure of the analysis chamber and the evaporation chamber were better than $5\times10^{-10}$and 5×10^{-9} Torr, respectively. The PT*b*T-Tos polymer films were obtained by spin-coating the solutions containing T*b*T monomers and Baytron C on 1 cm x 1 cm bare silicon wafer. After heating at 90 ^{o}C in air for 2 hours, the films were rinsed with ethanol three times and then transferred to hot plate at 90 ^{o}C heating for half an hour. The PT*b*T-Tos films were transferred to the analysis chamber without breaking the vacuum for UPS and XPS measurements. An unfiltered He-discharge lamp (21.22 eV) and a monochromatic Al Kα X-ray (1486.6 eV) excitation sources were respectively equipped for

UPS and XPS analysis. The energy resolution for UPS was 100 meV as estimated from the Fermi edge of an Ar^+ sputtered clean Au film. The samples were negatively biased at 9.0 V with respect to the electron analyzer for obtaining the secondary electron cutoff (SECO) spectra. The Fermi edge was calibrated from a UPS spectrum of the cleaned Au substrate. UV-VIS-NIR spectra of the polymer thin films were recorded on a JASCO V-570 spectrometer. The temperature dependent X-ray diffraction (XRD) measurements were measured using a Bruker D8 Advance diffractometer. The 2θ scanning range was set from 5° to 30° with step size of 0.02° and integration time of 2s. A Cu rotating anode was used as the X-ray source with the generator voltage set as 40 kV to maximize the incident light flux. A LYNXEYE XE energy-dispersive 1D detector with an array of 192 point detectors was employed to deliver superior signal/noise ratio. Synchrotron-based grazing-incidence X-ray diffraction (GIXD) were measured at the SAXS/WAXS beamline at the Australian Synchrotron. [2] 15 keV photons were used with scattering patterns recorded on a Dectris Pilatus 1M detector. Images shown were acquired at an incident angle close the critical angle. Such images were chosen from a series of images taken with incident X-ray angle varying from 0.02° to 0.15° in steps of 0.01° with the chosen image showing the highest scattering intesnity. The X-ray exposure time was 3 s such that no film damage was identified. The sample-to-detector distance as calibrated using a silver behenate sample. The results were analyzed by an altered version of the NIKA[3] 2D based in IgorPro.

Measurements of Thermoelectric Properties. The glass substrates were cleaned by distilled water, ethanol and acetone .The Au electrodes (30 nm) were then deposited by a shadow mask with a channel length of 500 μm and channel width of 5000 μm for Seebeck coefficient and conductivity measurement. The PT*b*T-Tos polymer films were obtained by spin-coating the solutions containing T*b*T monomers and Baytron C on the glass substrates. After heating at 90 ^{o}C in air for 2 hours, the films were rinsed with ethanol three times and then transferred to hot plate at 90 ^{o}C heating for half an hour. The electrical conductivity was tested by the four-

probe method with Keithley 4200 SCS. The Seebeck coefficient was measured in a vacuum chamber and calculated by the formula $S = V_{therm}/\Delta T$, where V_{therm} is the thermal voltage obtained by creating a temperature gradient (ΔT) at the two ends of the device by two Peliter elements and controlled by utilizing an infrared camera FLIR A300 (thermal sensitivity < 50 mK). The measurement of V_{therm} was conducted by Keithley 4200 SCS and the accuracy of the temperature measurements was verified by two resistive thermometers near the electrodes. The Seebeck coefficient and electrical conductivity were measured with the same device.[4]

2. Preparation and Characterization

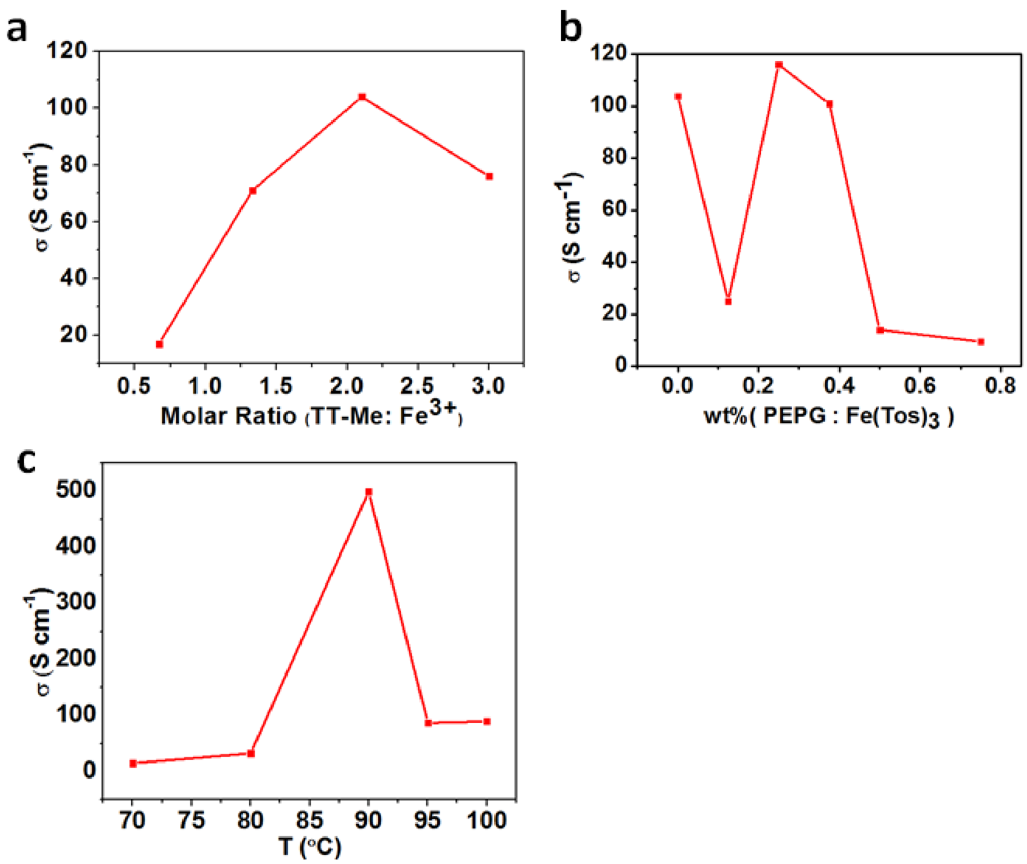

Figure S1. Optimized procedure: 600 mg of $Fe(Tos)_3$, 150 mg of PEPG, 41.48 mg of pyridine were added into *n*-Butanol solution (3 mL). The oxidative solution was stirred at room temperature for one day and a brown solution was obtained. T*b*T monomers (0.71 mol/L) with different alkyl groups were added to the oxidant and stirred for ten minutes. After that, the deep-brown mixtures were spin-coated on the corresponding substrates. (a) the molar ratio

of T*b*T monomer and $Fe(Tos)_3$; (b) the molar ratio of PEPG and $Fe(Tos)_3$; c) Polymerization temperature.

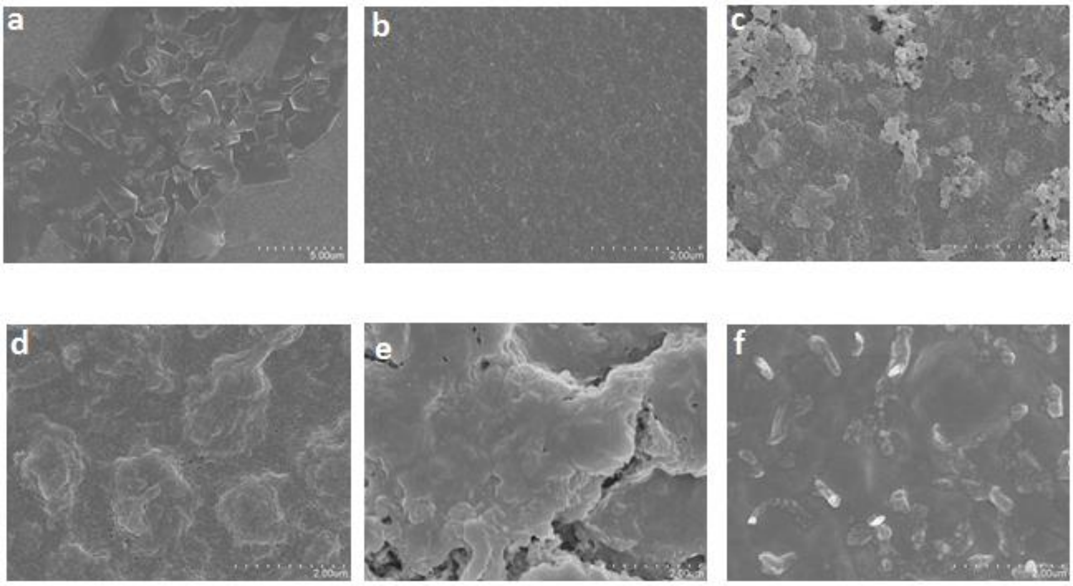

Figure S2. SEM images of a-f) PT*b*T-Tos-C0 to PT*b*T-Tos-C8 (bar: 2 μm).

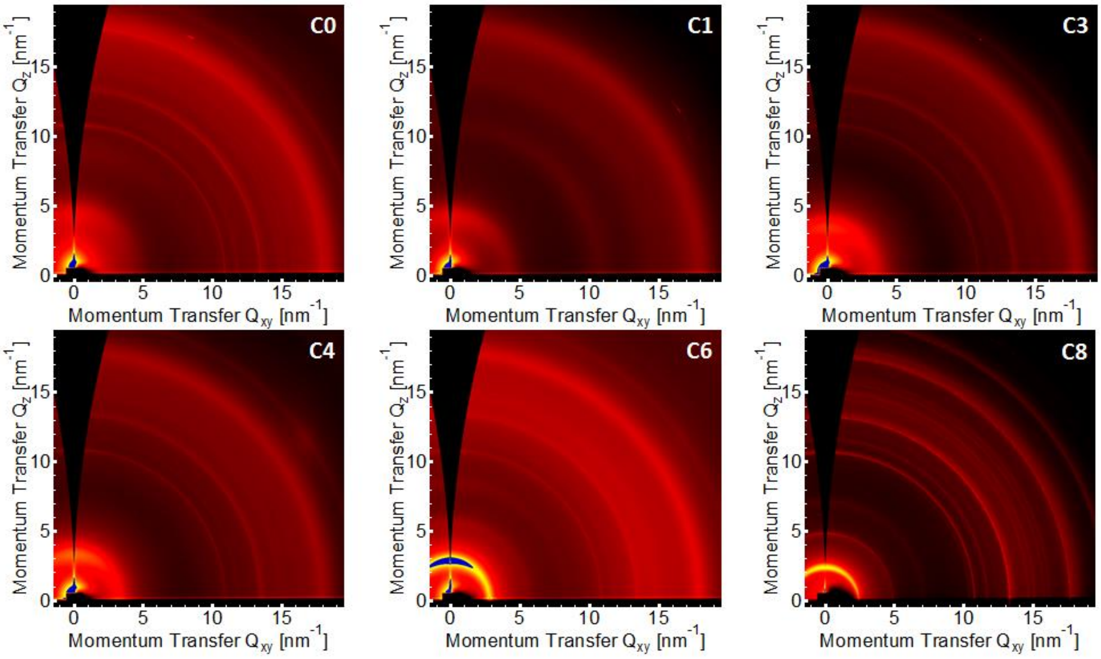

Figure S3. GIWAXS measurements of PT*b*T-Tos films with alky groups from C0 to C8.

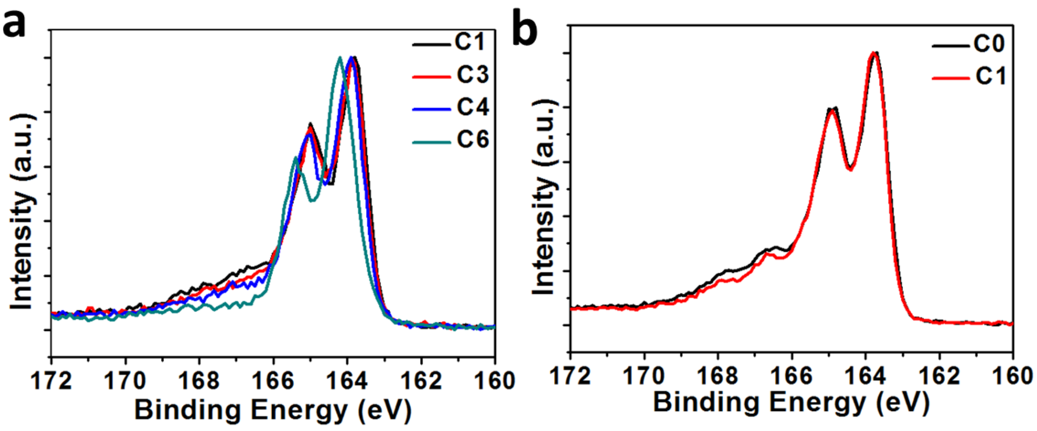

Figure S4. S (2p) XPS spectra of the PT*b*T-Tos polymers with alkyl group from C6 to C1 (a) and comparison of PT*b*T-C1-Tos and PT*b*T-C0-Tos (b).

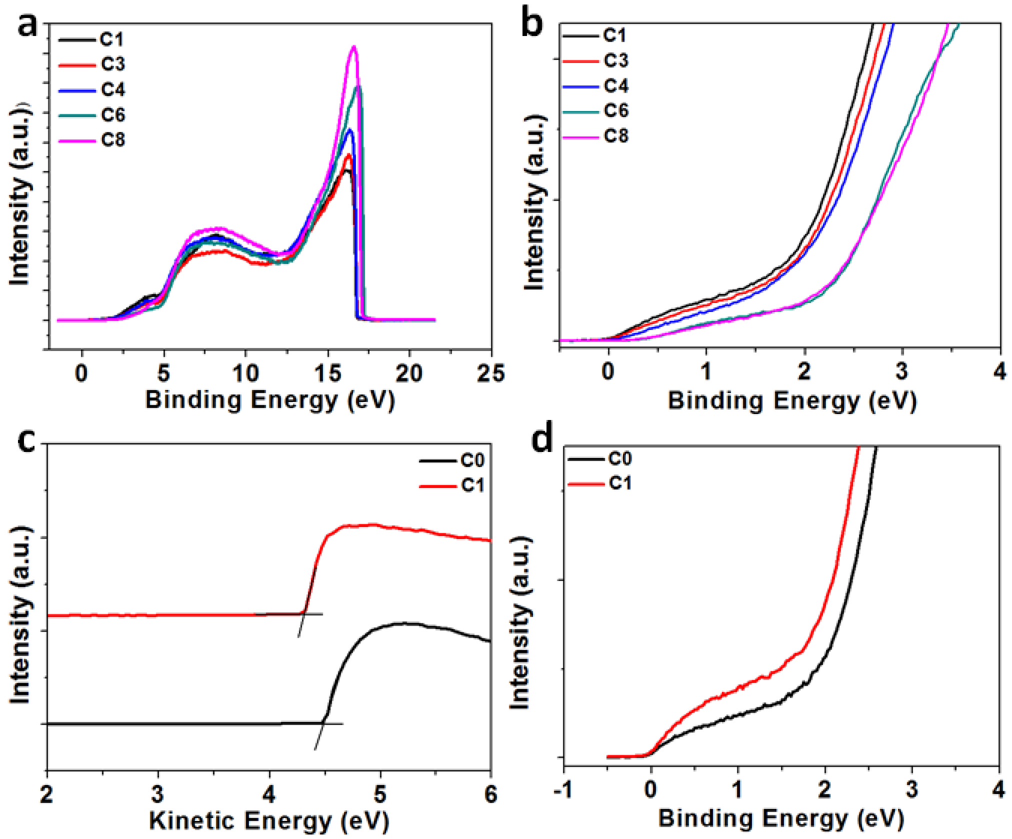

Figure S5. (a) UPS spectra and (b) low binding energy region (HOMO) from PT*b*T-Tos-C8 to PTbT-Tos-C1; The low kinetic energy region (c) and low binding energy region (HOMO) (d) of UPS spectra of PT*b*T-C1-Tos and PT*b*T-C0-Tos.

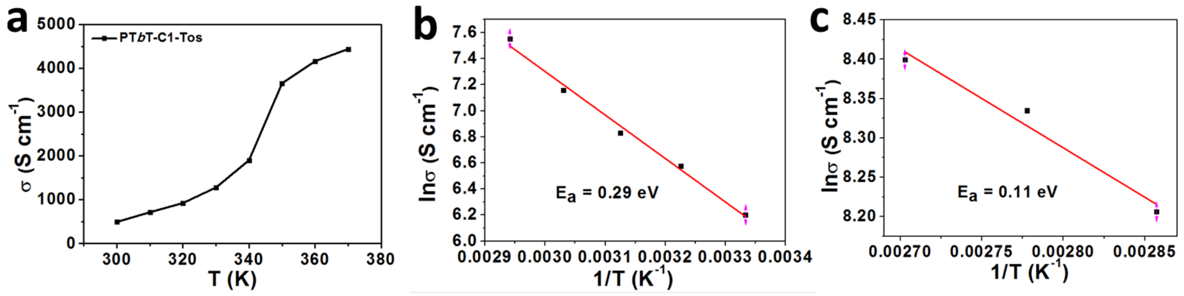

Figure S6. (a) Electrical conductivity changes with increasing temperature; ln σ vs 1/T of PT*b*T-C1-Tos fitting to Arrhenius equation at (b) low temperature region: 300-340K and (c) high temperature region: 350-370K.

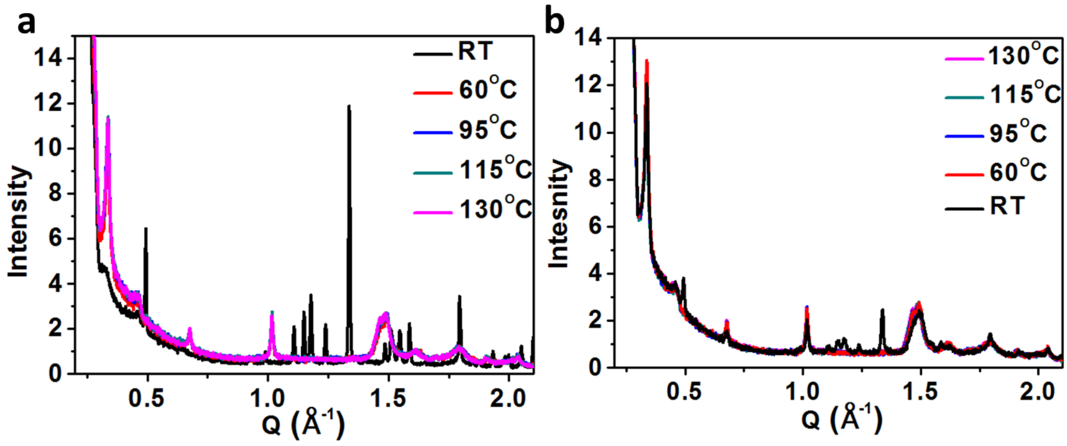

Figure S7. Temperature-variable XRD patterns of PT*b*T-C1-Tos bulk film. Left figure (a) shows changes in the XRD pattern with heating, while right figure (b) shows changes with cooling.

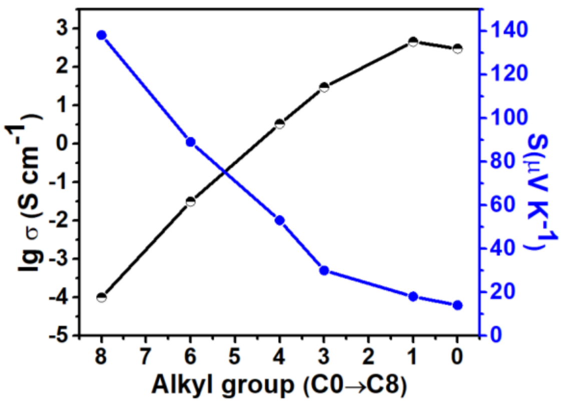

Figure S8. The electrical conductivity and Seebeck coefficients of PT*b*T-Tos with alkyl groups from C8 to C0.

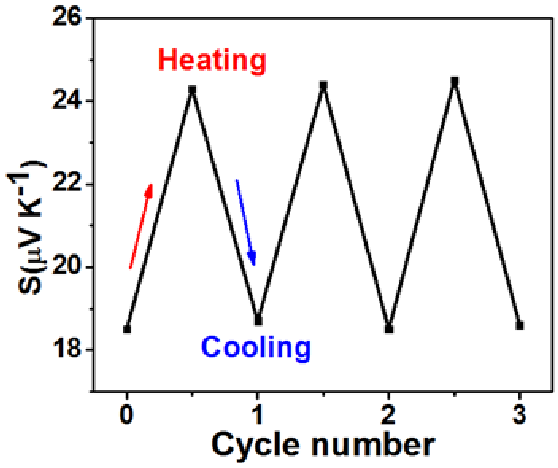

Figure S9. Seebeck coefficients of PT*b*T-C1-Tos film with heating and cooling (three cycles).

Table S1. Thermoelectric Performance of PT*b*T-C1-Tos and PEDOT-Tos prepared in this work.

	**σ (S cm^{-1})**	**S (μV K^{-1})**	**PF(μW m^{-1} K^{-2})**
**PT*b*T-C1-Tos**[a]	450	17	13.0
**PT*b*T-C1-Tos**[b]	4444	24	263
**PEDOT-Tos**[a]	600	10	6
**PEDOT-Tos**[b]	450	13	7.6

[a] Room temperature; [b] 373K.

As shown in Table S1, the optimized power factor of PT*b*T-C1-Tos at room temperature is around two times higher than that of PEDOT-Tos under the same conditions prepared in this work. The electrical conductivity of PEDOT-Tos (600 S cm^{-1}) is as high as the reported value, but the Seebeck coefficient is much lower (*Energy Environ. Sci.* **2013**, 6, 788.). The reason is unclear and might be attributed to the polymer preparation and measurement. Moreover, the power factor of PT*b*T-C1-Tos increased from 13.0 μW m^{-1} K^{-2} at room temperature to 263 μW m^{-1} K^{-2} at 370 K significantly, which is much higher than that of PEDOT-Tos, 7.6 μW m^{-1} K^{-2} at 370K.

3. References

[1] C. Zhang, Y. Zang, E. Gann, C. R. McNeill, X. Zhu, C. A. Di, D. Zhu, *J. Am. Chem. Soc.* **2014**, *136*, 16176.

[2] N. M. Kirby, S. T. Mudie, A. M. Hawley, D. J. Cookson, H. D. T. Mertens, N. Cowieson, V. Samardzic-Boban, *J. Appl. Crystallogr.* **2013**, *46*, 1670.

[3] J. Ilavsky, *J. Appl. Crystallogr.* **2012**, *45*, 324.

[4] D. Huang, C. Wang, Y. Zou, X. Shen, Y. Zang, H. Shen, X. Gao, Y. Yi, W. Xu, C.-a. Di, D. Zhu, *Angew. Chem., Int. Ed.* **2016**, *55*, 10672.
